# Oxidative Stress, Neuroinflammation and Neurodegeneration: The Chicken, the Egg and the Dinosaur

**DOI:** 10.3390/antiox11081554

**Published:** 2022-08-11

**Authors:** Peter M. J. Quinn, António Francisco Ambrósio, Celso Henrique Alves

**Affiliations:** 1 Department of Ophthalmology, Vagelos College of Physicians & Surgeons, Columbia University, New York, NY 10032, USA; 2 Coimbra Institute for Clinical and Biomedical Research (iCBR), Faculty of Medicine, University of Coimbra, 3000-548 Coimbra, Portugal; 3 Center for Innovative Biomedicine and Biotechnology (CIBB), University of Coimbra, 3004-531 Coimbra, Portugal; 4 Association for Innovation and Biomedical Research on Light and Image (AIBILI), 3000-548 Coimbra, Portugal; 5 Clinical Academic Center of Coimbra (CACC), 3004-561 Coimbra, Portugal

Neurodegenerative diseases are characterized by the progressive degeneration of the neuronal cells and their networks, hampering the function of the central or peripheral nervous system. Neurodegenerative diseases are a heterogeneous group of disorders that might affect different tissues, such as the brain, retina or spinal cord. This group can include diseases such as Alzheimer’s disease (AD), amyotrophic lateral sclerosis (ALS), multiple sclerosis (MS), Parkinson’s disease (PD), spinal muscular atrophy, spinocerebellar ataxias, age-related macular degeneration (AMD), glaucoma, retinitis pigmentosa (RP) or diabetic retinopathy (DR) [1]. Five of these diseases alone cumulatively account for more than 401 million affected people worldwide—Alzheimer’s disease (32 million) [2], Parkinson’s disease (6.1 million) [3], age-related macular degeneration (196 million) [4], glaucoma (64.3 million) [5], diabetic retinopathy (103.12 million) [6]—and these numbers will continue to grow in the coming decades, foretelling a heavy public health, economical and societal burden.

Neurodegeneration in these diseases seems to be tightly linked to increased oxidative stress and neuroinflammation [7]. However, answering the question of what among oxidative stress, neuroinflammation and neurodegeneration initiates and contributes the most to the pathophysiology of neurodegenerative disorders is very similar to asking who was born first—the chicken or the egg? The answer seems to be far more complex than simply answering the chicken or the egg. It appears that neither oxidative stress nor inflammation or neurodegeneration are by themselves the main component or driver of these diseases. In the end, the answer might become unexpected, and we might end up with a dinosaur—an egg-laying creature preceding birds in the evolutionary tree. It is now becoming clear that what is essential is a fine-tuned balance between the three processes.

Thus, understanding the link between oxidative stress, neuroinflammation and neurodegeneration processes is crucial to defining preventive and interventive measures and developing new therapies for these devastating diseases. This “Oxidative stress in Neurodegeneration and Neuroinflammation” Special Issue aimed to contribute toward clarifying these questions and gather the most recent findings on the role of oxidative stress and its relationship with neuroinflammation and neurodegeneration.

The “Oxidative stress in Neurodegeneration and Neuroinflammation” Special Issue comprises seven review articles and six original research articles. Among the review articles, three of them focused on the involvement of oxidative stress in eye disorders, such as vitreoretinal diseases [8], diseases associated with retinal ganglion cells degeneration [9] and on the role of bisretinoids of the retina in photo-oxidation, iron-catalyzed oxidation and disease consequences [10]. The other four review papers summarized the major causes of central nervous system (CNS) redox homeostasis imbalance [11], the significance of amyloid β-protein oligomers (AβOs) and oxidative stress in AD [12], the role of TRAP1 in oxidative stress and neurodegeneration [7] and how PON2 controls oxidative stress, inhibits apoptosis and contributes to the progression of various types of malignancies [13].

Novel pathogenic mechanisms and therapeutic approaches for eye disorders were described here, holding hope for the development of new treatments for optic neuropathies, based on intravitreal injections of pegylated granulocyte colony-stimulating factor (G-CSF) [14], and for AMD patients carrying Y402H polymorphism in the factor H protein (FH), based on the mTOR inhibition [15]. Moreover, it was demonstrated here that the redox-sensitive protein DJ-1 is required to protect the retina and retinal pigment epithelium (RPE) from oxidative-stress-induced degeneration [16]. This Special Issue also gathered studies focused on the identification of new biomarkers for AD preclinical diagnosis [17], of new treatments based on the administration of hydroxocobalamin (Hb, vitamin B_12_ analog) to prevent cytoplasmic aggregation of TDP-43 observed in many neurodegenerative diseases [18], of a new mechanism linking astrocyte-derived oxidative stress to motor-neuron damage in ALS [19], and on the role of the alpha–ketoglutarate dehydrogenase complex (KGDHC), a rate-limiting enzyme in the tricarboxylic acid cycle, whose activity is strikingly reduced in AD [16], in the bioenergetics and reactive oxygen species (ROS) homeostasis of brain mitochondria [20].

The works gathered in this Special Issue will certainly help clarify the role of oxidative stress in neuroinflammatory and neurodegenerative processes. This valuable new knowledge will contribute to unravelling new disease pathways, new diagnostic tools and new therapies for such devastating disorders.
